# Comparison of two self-reported tools for assessment of sleep quality

**DOI:** 10.1080/1179271X.2026.2724201

**Published:** 2026-09-06

**Authors:** Melissa J. Benton, Andrea M. Hutchins

**Affiliations:** ^a^ Department of Nursing, Helen and Arthur E. Johnson Beth-El College of Nursing & Health Sciences, University of Colorado Colorado Springs, Colorado Springs, USA; ^b^ Department of Human Physiology & Nutrition, Helen and Arthur E. Johnson Beth-El College of Nursing & Health Sciences, University of Colorado Colorado Springs, Colorado Springs, USA

**Keywords:** Richards–Campbell Sleep Questionnaire, Restorative Sleep Questionnaire, health-related quality of life, well-being, perceived stress, sleep quality

## Abstract

**Objective:**

An easy to administer tool is needed for clinical assessment of sleep quality by healthcare providers. This study compared two brief, validated sleep questionnaires in order to identify an appropriate tool for clinical assessment.

**Methods:**

Women (N = 121) between the ages of 25–89 years (60.3 ± 15.9 years) completed the Richards-Campbell Sleep Questionnaire, the Restorative Sleep Questionnaire, and associated measures of quality of life, well-being, stress, and physical activity.

**Results:**

There were no differences in sleep quality assessments between the two questionnaires for sleep last night (daily), during the past 7 days (weekly), or during the past 4 weeks (monthly). Overall, participants reported good daily, weekly, and monthly sleep quality (mean scores 71.1–75.3 out of 100), although both questionnaires assessed significantly better (*p* ≤ 0.002) daily sleep quality compared to weekly and monthly. There were no floor effects for either questionnaire, but ceiling effects were noted for the Richards-Campbell Sleep Questionnaire daily and weekly versions. When participants were grouped by sleep quality, very good sleepers reported better quality of life (*p*˂ 0.05) and well-being (*p*˂ 0.001), and less stress (*p*˂ 0.05) compared to poor sleepers for all versions of the Restorative Sleep Questionnaire and for the Richards-Campbell Sleep Questionnaire daily and weekly versions.

**Conclusions:**

For overall identification and assessment of good versus poor sleep quality, clinicians can choose either the Richards-Campbell Sleep Questionnaire or the Restorative Sleep Questionnaire. However, caution may be needed for short-term (daily, weekly) assessment for which the Restorative Sleep Questionnaire may be more appropriate due to fewer ceiling effects.

## PLAIN LANGUAGE SUMMARY

Good sleep quality improves health and quality of life. By comparison, poor sleep quality increases the risk for disease and premature death. Women are more likely than men to experience poor sleep quality. Healthcare providers need a brief, easy to use tool to accurately measure sleep quality as part of health assessment. Unfortunately, sleep quality is a complex concept and numerous tools are available. To identify an appropriate tool for clinical practice, we compared two short questionnaires that measure different aspects of sleep quality. A group of 121 healthy women living in the community completed daily, weekly, and monthly versions of the Richards-Campbell Sleep Questionnaire (RCSQ) and the Restorative Sleep Questionnaire (RSQ). To standardize scores, a 5-point scale was used for both tools. The RCSQ and RSQ measured sleep quality the same, although women reported better daily sleep than weekly and monthly sleep using both tools. When women were grouped into good and poor sleepers, the daily and weekly versions of both tools accurately discriminated differences in stress, well-being, and quality of life. We also assessed the number of women who reported the maximum possible sleep score (ceiling effect). A large number of women reported the highest possible sleep score using the RCSQ for daily and weekly assessment, which may be a potential problem with this tool. Overall, both tools provide comparable and accurate measurement of sleep quality, but some caution may be needed for daily and weekly assessment, for which the RSQ may be more appropriate.

## Introduction

Sleep is associated with multiple health benefits. Good sleep quality improves mental health, including decreased depression, anxiety, and stress,^1^ and is associated with better self-reported quality of life.^2^^,^^3^ In middle-aged and older adults, better sleep quality is associated with decreased risk for obesity,^2^^,^^4^^,^^5^ and among older adults, improved sleep quality is associated with improved muscle strength and function.^6^ Among breast cancer survivors, better sleep quality predicts longer survival.^7^

By comparison, poor sleep quality is associated with increased inflammatory markers, impaired cognitive performance, and decreased pain tolerance,^8–11^ as well as increased risk for chronic diseases such as hypertension, diabetes, and heart disease.^12^^,^^13^ Among adults with inflammatory bowel disease, impaired sleep increases risk for adverse health outcomes including surgery, hospitalization, and disease progression,^14^ and among those with chronic kidney disease, poor sleep is associated with decreased renal function.^15^ Increased age is associated with decreased sleep quality in healthy adults,^16^ and in older men and women, poor sleep quality can increase mortality risk by more than 25%.^17^ Furthermore, mortality risk related to cardiovascular disease is more than three times greater among women with poor sleep quality.^18^

The concept of sleep quality is complex and perceptions of good sleep quality are often based on sleep continuity or duration,^19–22^ including both total sleep time and sleep efficiency.^16^^,^^23^ Restorative sleep is another characteristic of sleep that has been described as the outcome of good quality sleep.^11^^,^^24^^,^^25^ Although healthy adults should sleep at least seven hours per night,^26^ the global prevalence of poor sleep is reported to be as high as 40% among community-dwelling older adults,^27^ and can exceed 50% among adults with chronic diseases.^28–30^ Furthermore, there is convincing evidence that women experience poorer sleep quality compared to men.^31^^,^^32^

Beginning in adolescence, women report an increasing prevalence of insomnia and poor quality sleep compared to men.^31^ Although there is evidence that women perceive poorer subjective sleep quality compared to their objectively measured sleep quality,^33^ there is also evidence confirming their reports of poor sleep quality with objective measurement using polysomnography.^34^^,^^35^ Among women, reductions in sleep quality have been attributed to hormonal fluctuations related to the menstrual cycle,^36^ as well as the hormonal changes that occur during menopause.^37^ At the time of menopause, as many as 60% of women report poor sleep quality.^38^ Furthermore, risk factors such as stress, physical inactivity, and current smoking have been found to be associated with poor sleep quality among women.^39–41^

Regular clinical assessment of sleep by healthcare providers has been recommended in order to identify poor sleepers and implement health promotion interventions.^24^^,^^42^^,^^43^ However, clinical assessment tools should be brief and easy to administer.^24^ The most frequently reported tool for subjective measurement of sleep quality in research studies is the Pittsburgh Sleep Quality Index (PSQI).^20^^,^^44^ The PSQI includes 19 self-reported items plus 5 items rated by a roommate or bed partner that require 5–10 minutes for respondents to complete.^20^^,^^45^ However, scoring for the PSQI is complex and awkward,^44^ which creates a challenge for efficient use in clinical settings. Furthermore, specific populations, such as older adults, can have difficulty interpreting the PSQI items, limiting its accuracy.^46^

A brief, easy to administer tool is needed for rapid clinical assessment of sleep quality. There are a number of self-reported questionnaires developed for community and hospital settings that assess patient perceptions of the sleep state (direct sleep quality), as well as the restorative nature of sleep (feelings of restfulness or being refreshed).^47^^,^^48^ Due to the complexity of the concept of sleep quality, it is unclear which construct is most appropriate for clinical measurement. Therefore, a comparison of two validated and well-known tools that measure either perceptions of sleep or the restorative nature of sleep and have been validated for community use would provide useful guidance for clinicians.

The Richards-Campbell Sleep Questionnaire^49^ and the Restorative Sleep Questionnaire^50^ are both brief (less than 10 items each) and easy to administer self-reported tools. The Richards-Campbell Sleep Questionnaire, which assesses the direct perception of sleep, has been used primarily with critically ill hospitalized populations,^48^^,^^51–54^ although it has also been validated in community-dwelling adults.^52^ By comparison, the Restorative Sleep Questionnaire, which assesses the restorative nature of sleep, has been used solely with healthy community-dwelling populations.^55–58^ The Richards-Campbell Sleep Questionnaire can be completed in approximately 2 minutes.^53^ Although we can find no report of the time needed for completion of the Restorative Sleep Questionnaire, it seems certain that with less than 10 items in the questionnaire, less than 5 minutes would be needed.

The Richards-Campbell Sleep Questionnaire and Restorative Sleep Questionnaire provide feasible alternatives for sleep assessment in busy clinical settings but have not yet been compared in a single study, nor have potential floor and ceiling effects been explored. Therefore, the purpose of this study was to provide a comparison that will allow healthcare providers to identify an appropriate tool for rapid assessment of sleep quality. Based on gender differences in sleep quality, only women were recruited for the current study.

## Methods

A total of 121 women volunteered to participate in this cross-sectional study. Women were included if they were at least 25 years old and current non-smokers. All testing occurred in a single university laboratory with only the participant and investigator present. To control for potential circadian rhythm effects on perceived sleep quality, all testing was completed in the morning. The study was approved by the Institutional Review Board of the University of Colorado Colorado Springs and all participants gave written informed consent prior to inclusion.

### Assessments

#### Sleep quality

The Richards-Campbell Sleep Questionnaire and Restorative Sleep Questionnaire were used to assess sleep quality. Three versions of each questionnaire were completed in random order asking participants about their sleep “last night,” “during the past 7 days,” and “during the past 4 weeks.”

The Richards-Campbell Sleep Questionnaire is a 5-item tool that has been validated against polysomnography,^49^^,^^59^ and also found to be correlated with actigraphy.^60^^,^^61^ Conceptually, it characterizes perceptions of the immediate sleep experience, and was originally developed and validated to assess sleep “last night” using a 100 mm visual analog scale (VAS) for scoring.^49^ Items assess sleep depth, falling asleep, awakening, time awake, and sleep quality, with anchor terms for each item placed at either end of the VAS (e.g. deep sleep – light sleep). Scores for each item must be calculated by physically measuring the distance in mm across the VAS, which may be inefficient in a busy clinical setting. For the current study, we chose to assign values from 1 to 5 to corresponding points on the VAS to facilitate scoring and for consistency with the Likert-scale structure used in the Restorative Sleep Questionnaire. Scores for each item were summed and divided by five to calculate an average total sleep score ranging from 1 to 5, with higher scores indicating better sleep. Although small differences in scores between VAS and Likert-like scales have been found, they were not statistically significant and use of a Likert scale has been recommended due to its ease of administration and scoring.^62^ For this study, total scores were converted to a 0–100 scale using the formula described for the Restorative Sleep Questionnaire: [(total sleep score – 1) × 25].^50^ Total scores ≤25 were defined as very poor sleep and total scores ≥76 were defined as very good sleep.^63^ Scale reliability (Cronbach’s alpha) for the Richards-Campbell Sleep Questionnaire was originally reported to be 0.90.^49^ For the current study, Cronbach’s alpha was 0.90 for sleep last night, 0.91 for sleep during the past 7 days, and 0.91 for sleep during the past 4 weeks.

The Restorative Sleep Questionnaire is a 9-item tool that has been validated against polysomnography.^50^ Conceptually, it defines restorative sleep as a feeling of being refreshed after waking.^50^ Two versions were originally developed and validated to assess sleep “each day” (daily) and “during the past 7 days” (weekly). The questionnaire asks respondents to rate their feelings after awakening using a five-point Likert-scale (1 – not at all, 2 – a little bit, 3 – some; 4 – very much, 5 – completely). Six items regarding positive characteristics (good mood, rested, refreshed/restored, ready to start the day, energetic, mentally alert) are directly scored, and three items regarding negative characteristics (tired, sleepy, grouchy) are reverse scored. Consistent with recommended scoring guidelines,^50^ scores for each item were summed and divided by nine to calculate an average total sleep score ranging from 1 to 5, with higher scores indicating better sleep quality. Total scores were converted to a 0–100 scale using the formula: [(total sleep score – 1) × 25].^50^ Total scores ≤25 were defined as very poor sleep and total scores ≥76 were defined as very good sleep.^63^ Scale reliability (Cronbach’s alpha) for the Restorative Sleep Questionnaire was originally reported to be 0.91 for the daily version and 0.90 for the weekly version.^50^ For the current study, Cronbach’s alpha was 0.91 for sleep last night, 0.90 for sleep during the past 7 days, and 0.91 for sleep during the past 4 weeks.

#### Comparison measures

The RAND-36 is a validated measure of health-related quality of life that is open-access and includes the same items as the SF-36.^64^^,^^65^ Thirty-six items assess physical and mental components of quality of life, including subscales for physical function, physical role limitations, vitality, emotional well-being, emotional role limitations, social function, pain, and general health. Scores are calculated ranging from 1 to 100, with higher scores indicating better quality of life. The Restorative Sleep Questionnaire has previously been validated against the SF-36 and found to have the strongest correlations (daily = 0.66, weekly = 0.82) with the vitality subscale that assesses how respondents feel “during the past 4 weeks”.^50^ For the current study, RAND-36 vitality subscale scores were used for analysis. Cronbach’s alpha for the RAND-36 vitality subscale was originally reported to be 0.86.^64^ For the current study, Cronbach’s alpha for the vitality subscale was 0.84.

The Vitality Plus Scale (VPS) is a 10-item tool that assesses current feelings of well-being. The VPS has been validated against the SF-36 and found to have a correlation of .65 with the SF-36 vitality subscale.^66^ Its structure is similar to that of the Richards-Campbell Sleep Questionnaire. Individual items assess 10 aspects of well-being that include time to fall asleep, sleep quality, feeling drowsy, hunger, pain, and energy levels, with anchor terms for each item at either end of a 5-point scale (e.g. takes a long time to fall asleep – fall asleep quickly). Scores for each item are summed to calculate a total score ranging from 10 to 50, with higher scores indicating greater well-being. For the current study, VPS total scores and individual scores for three items regarding time to fall asleep (long time – quickly), sleep quality (very poorly – well), and feelings during the day (drowsy – rested) were used for analysis. Cronbach’s alpha for the VPS was originally reported to be 0.81.^66^ For the current study, Cronbach’s alpha for the VPS was 0.78.

The Perceived Stress Scale (PSS-10) is a 10-item tool that measures the extent to which life events are perceived as stressful “during the last month”.^67^ Item responses use a 5-point Likert scale (0 – never, 1 – almost never, 2 – sometimes, 3 – fairly often, 4 – very often). Six negatively worded items are directly scored and four positively worded items are reverse scored. Scores for each item are summed to calculate a total score ranging from 0 to 40, with higher scores indicating greater perceived stress. The PSS-10 has been previously used for research with both the Restorative Sleep Questionnaire^57^^,^^68^ and the Richards-Campbell Sleep Questionnaire.^69^ In all three studies perceived stress was found to be consistently associated with changes in sleep quality. Specifically, as perceived stress decreased, sleep quality was observed to increase.^57^^,^^68^^,^^69^ For the current study, PSS-10 total scores were used for analysis. Cronbach’s alpha for the PSS-10 was originally reported to be 0.78, and over multiple studies ranged from 0.74–0.91.^70^ For the current study, Cronbach’s alpha for the PSS-10 was 0.82.

The International Physical Activity Questionnaire – Short Form (IPAQ-SF) is a widely-recognized self-reported measure of physical activity that has been validated against actigraphy.^71^^,^^72^ Although neither the Richards-Campbell Sleep Questionnaire nor the Restorative Sleep Questionnaire have been validated against the IPAQ-SF, the Restorative Sleep Questionnaire has been used for research with actigraphy and found to be sensitive to changes in objectively measured activity.^55^^,^^73^ Therefore, an a priori decision was made to include the IPAQ-SF among the comparison variables based on the recognized relationship between sleep and physical activity.^74–76^ The IPAQ-SF includes 6 items assessing the frequency and duration of walking, moderate intensity activity, and vigorous intensity activity “during the past seven days.” Scores are calculated as: [(number of days X minutes of activity) × the MET value for each type of activity]. Metabolic equivalent (MET) values are 3.3 for walking, 4.0 for moderate intensity, and 8.0 for vigorous intensity. The three category scores are summed to calculate a total activity score measured as MET-min/week. Low activity is defined as less than 600 MET-min/week. Moderate activity is defined as at least 600 MET-min/week. High activity is defined as at least 3000 MET-min/week.^77^ For the current study, MET-min/week for individual activity levels (walking, moderate, and vigorous), as well as the three activity categories (low, moderate, and high) were used for analysis.

#### Statistical analysis

Data were analyzed using SPSS version 31. Descriptive statistics were used to analyze participant characteristics and data were reported as means ± standard deviations or frequencies (percents). Statistical significance was pre-determined as *p* ˂ 0.05. Sleep quality scores were not normally distributed, so non-parametric tests were used for comparative analyses and to evaluate relationships. Spearman rank correlations were used to evaluate relationships and Kruskal–Wallis H tests and Wilcoxon signed-rank tests were used to compare differences. Floor and ceiling effects were determined to exist for the two sleep quality measures if more than 15% of participants reported the lowest (0 points) or highest (100 points) possible scores.^78^^,^^79^

## Results

Overall, participants ranged in age from 25 to 89 years, with an average age of 60 ± 15.9 years (Table 1). The majority self-identified as White (82%), followed by Black (7%) and Hispanic (7%). Daily, weekly, and monthly mean sleep scores for both the Richards-Campbell Sleep Questionnaire and the Restorative Sleep Questionnaire consistently reflected good quality sleep. Approximately half (49%) of the participants reported high levels of physical activity and 40% reported moderate levels of activity.

**Table 1.** Participant characteristics.

	Mean ± SD	Frequency (%)
Age (years)	60.3 ± 15.9	
Race/Ethnicity		
White		99 (82)
Black		8 (7)
Hispanic		9 (7)
Asian		1 (1)
Hispanic/Black		2 (2)
Native American		1 (1)
Body weight (kg)	71.9 ± 15.7	
Body mass index (kg/m^2^)	27.2 ± 5.9	
Waist circumference (cm)	88.5 ± 13.3	
Sleep Quality scores		
RCSQ		
Day	75.3 ± 23.9	
Week	73.4 ± 22.1	
Month	71.1 ± 22.2	
RSQ		
Day	75.2 ± 18.8	
Week	72.8 ± 17.3	
Month	71.9 ± 18.6	
RAND-36 Vitality subscale	63.0 ± 18.4	
Vitality Plus Scale	36.0 ± 6.9	
Perceived Stress Scale	10.7 ± 5.9	
IPAQ-SF (MET-min/week)		
Vigorous intensity	1544.3 ± 1871.4	
Moderate intensity	1252.3 ± 1447.2	
Walking	1466.2 ± 1586.1	
Activity levels		
High		59 (49)
Moderate		48 (40)
Low		11 (9)

Abbreviations. RCSQ, Richards-Campbell Sleep Questionnaire; RSQ, Restorative Sleep Questionnaire; IPAQ-SF, International Physical Activity Questionnaire-Short Form.

### Sleep quality

There were no significant between-group differences in sleep quality scores between the Richards-Campbell Sleep Questionnaire and the Restorative Sleep Questionnaire for daily, weekly, or monthly time periods. Participants reported similar median values for sleep quality using both tools (Table 2). However, statistically significant within-group differences were reported for sleep quality over the three time periods. Sleep quality last night (daily) was significantly better (*p* = .002) than sleep quality during the past 7 days (weekly) and past 4 weeks (monthly) when assessed with the Restorative Sleep Questionnaire. When assessed with the Richards-Campbell Sleep Questionnaire, sleep quality for last night and the past 7 days was significantly better (*p* ˂ 0.001) than for the past 4 weeks. However, when participants were grouped by sleep quality (very poor, poor, good, very good) there were no between-group differences in frequencies as assessed by the two questionnaires. Approximately 50% of participants reported very good sleep quality last night (daily) and during the past 7 days (weekly), while only approximately 40% reported very good sleep during the past 4 weeks (monthly). By comparison, no more than 5% reported very poor sleep quality for any time period. Due to the low number of participants reporting very poor and poor quality sleep, the two categories were collapsed to form three sleep quality groups (poor, good, very good) for further comparison.

**Table 2.** Between and within-group comparisons of Richards-Campbell Sleep Questionnaire and Restorative Sleep Questionnaire scores for daily, weekly, and monthly sleep quality.

	RCSQ median (IQR)	RSQ median (IQR)	Between group *P*-value
Sleep last night (daily)	80 (55–100)** ^**	77.5 (60–92.5)* ^	0.988
Sleep past 7 days (weekly)	75 (60–95)** ^**	75 (57.8–83.8)	0.824
Sleep past 4 weeks (monthly)	75 (60–90)	75 (58.8–85)	0.539
**Within Group *P*-value**	**˂0.001**	**0.002**	
	Frequency (%)	Frequency (%)	
Sleep Quality (daily)			.124
Very poor	6 (5)	1 (1)	
Poor	17 (14)	14 (12)	
Good	35 (29)	37 (31)	
Very good	63 (52)	69 (57)	
Sleep Quality (weekly)			.295
Very poor	5 (4)	1 (1)	
Poor	17 (14)	16 (13)	
Good	44 (36)	45 (37)	
Very good	55 (46)	59 (49)	
Sleep Quality (monthly)			.200
Very poor	5 (4)	0	
Poor	21 (17)	17 (14)	
Good	46 (38)	52 (43)	
Very good	49 (41)	53 (43)	

Abbreviations. RCSQ, Richards-Campbell Sleep Questionnaire; RSQ, Restorative Sleep Questionnaire.

Data reported as Median (IQR) or Frequency (%).

* Significantly different from weekly.

^ Significantly different from monthly.

### Floor and ceiling effects

No floor effects were observed for either the Richards-Campbell Sleep Questionnaire or the Restorative Sleep Questionnaire. Ceiling effects were observed for the Richards-Campbell Sleep Questionnaire daily and weekly versions only. Twenty-six percent of participants scored 100 for sleep last night and 18% scored 100 for sleep during the past 7 days. No ceiling effects were noted for the Restorative Sleep Questionnaire.

### Health-related quality of life

Moderate positive correlations (Table 3) were found between the Restorative Sleep Questionnaire and health-related quality of life measured with the RAND-36 vitality subscale for daily, weekly, and monthly sleep. By comparison, the Richards-Campbell Sleep Questionnaire was found to have only a weak relationship with health-related quality of life specifically for weekly sleep, while daily and monthly sleep were not found to be related. When sleep quality groups were compared using the Richards-Campbell Sleep Questionnaire (Table 4), very good sleepers reported significantly better quality of life compared to poor sleepers for daily (*p* = 0.048) and weekly (*p* = 0.025) sleep, but no differences were observed for monthly sleep. When sleep quality groups were compared using the Restorative Sleep Questionnaire, very good sleepers reported significantly better quality of life compared to poor sleepers for daily, weekly, and monthly sleep (all *p* ˂ 0.001).

**Table 3.** Correlations between sleep quality and health-related quality of life (RAND-36 vitality subscale), the Vitality Plus scale, and the Perceived Stress scale.

	RAND-36 vitality subscale	Vitality plus Scale	*Time falling asleep	*Sleep quality	*Feelings during day	Perceived stress scale
RCSQ-Day	0.134 0.144	**0.394** ˂0.001	**0.453** ˂0.001	**0.573** ˂0.001	**0.235** 0.009	**−0.189** 0.038
RCSQ-Week	**0.179** 0.049	**0.507** ˂0.001	**0.603** ˂0.001	**0.651** ˂0.001	**0.330** ˂0.001	**−0.286** 0.001
RCSQ-Month	0.156 0.087	**0.447** ˂0.001	**0.600** ˂0.001	**0.592** ˂0.001	**0.341** ˂0.001	**−0.247** 0.006
RSQ-Day	**0.551** ˂0.001	**0.417** ˂0.001	**0.236** 0.009	**0.241** 0.008	**0.460** ˂0.001	**−0.521** ˂0.001
RSQ-Week	**0.566** ˂0.001	**0.357** ˂0.001	**0.300** ˂0.001	0.162 0.075	**0.362** ˂0.001	**−0.511** ˂0.001
RSQ-Month	**0.585** ˂0.001	**0.348** ˂0.001	**0.269** 0.003	0.132 0.148	**0.399** ˂0.001	**−0.571** ˂0.001

Abbreviations. RCSQ, Richards-Campbell Sleep Questionnaire; RSQ, Restorative Sleep Questionnaire.

Data reported as Spearman’s rho (*r*_s_); *p*-value. Correlation values in bold are statistically significant.

* Single items from Vitality Plus Scale.

**Table 4.** Comparison of health-related quality of life (RAND-36 vitality), Vitality Plus scale, and Perceived Stress scale by sleep quality groups (poor, good, very good) for (A) sleep last night, (B) sleep during the past 7 days, and (C) sleep during the past 4 weeks.

A	RCSQ-Day				RSQ-Day			
	Poor (n = 23)	Good (n = 35)	Very good (n = 63)	Within group	Poor (n = 15)	Good (n = 37)	Very good (n = 69)	Within group
RAND-36 Vitality Subscale	53.5 ± 20.14* ^	66.7 ± 14.7	64.4 ± 18.8	*H*(2) = 6.065 **0.048**	48.7 ± 18.1** ^**	54.2 ± 16.7** ^**	70.8 ± 15.4	*H*(2) = 30.091 **˂0.001**
Vitality Plus Scale	31.8 ± 6.7** ^**	35 ± 6.1** ^**	38 ± 6.7	*H*(2) = 16.095 **˂0.001**	31.3 ± 6.4** ^**	33.5 ± 6.7** ^**	38.3 ± 6.2	*H*(2) = 21.793 **˂0.001**
Perceived Stress Scale	14 ± 6.7** ^**	10.8 ± 5.7	9.9 ± 5.3	*H*(2) = 7.887 **0.019**	13 ± 5.7** ^**	14.5 ± 6.1** ^**	8.2 ± 4.5	*H*(2) = 28.366 **˂0.001**
**B**	RCSQ-Week				RSQ-Week			
	Poor (n = 22)	Good (n = 44)	Very Good (n = 55)	Within Group	Poor (n = 17)	Good (n = 45)	Very Good (n = 59)	Within Group
RAND-36 Vitality Subscale	53.4 ± 18.8* ^	63.2 ± 16.4** ^**	66.6 ± 18.8	*H*(2) = 7.375 **0.025**	45 ± 18.5* ^	59 ± 16** ^**	71.2 ± 15.5	*H*(2) = 28.540 **˂0.001**
Vitality Plus Scale	30.2 ± 5.7* ^	35.2 ± 5.4** ^**	38.9 ± 6.8	*H*(2) = 29.865 **˂0.001**	31.8 ± 7.4** ^**	34.6 ± 6.2** ^**	38.3 ± 6.5	*H*(2) = 15.404 **˂0.001**
Perceived Stress Scale	14.4 ± 5.8** ^**	11 ± 5.9	9 ± 5.4	*H*(2) = 11.997 **0.002**	15.8 ± 6.4** ^**	12.6 ± 5.7** ^**	7.9 ± 4.2	*H*(2) = 29.048 **˂0.001**
**C**	RCSQ-Month				RSQ-Month			
	Poor (n = 26)	Good (n = 46)	Very good (n = 49)	Within group	Poor (n = 17)	Good (n = 52)	Very good (n = 52)	Within group
RAND-36 Vitality Subscale	58.7 ± 19.1	61.5 ± 18.9	66.6 ± 17.3	*H*(2) = 2.484 .289	44.12 ± 20.3^	59 ± 16.1** ^**	73.1 ± 13.1	*H*(2) = 31.947 **˂.001**
Vitality Plus Scale	31.5 ± 6.0* ^	35.8 ± 5.5** ^**	38.5 ± 7.4	*H*(2) = 21.821 **˂.001**	33.8 ± 7.1** ^**	34.2 ± 6.4** ^**	38.5 ± 6.6	*H*(2) = 14.120 **˂.001**
Perceived Stress Scale	12.9 ± 6.2	11.1 ± 6	9.3 ± 5.4	*H*(2) = 5.733 .057	14.7 ± 5.9** ^**	12.9 ± 5.7** ^**	7.2 ± 4	*H*(2) = 34.424 **˂.001**

Abbreviations. RCSQ, Richards-Campbell Sleep Questionnaire; RSQ, Restorative Sleep Questionnaire.

Data reported as mean ± standard deviation; Kruskal-Wallis *H*-statistic; *p*-value. P-values in bold are statistically significant.

* Significantly different from Good.

^^^ Significantly different from Very Good.

### Well-being

Moderate positive correlations (Table 3) were found between the Richards-Campbell Sleep Questionnaire and the VPS total score for weekly and monthly sleep quality, while a weak positive correlation was found for daily sleep. By comparison, positive correlations with the Restorative Sleep Questionnaire were moderate only for daily sleep, and were weak for both weekly and monthly sleep quality. When sleep quality groups were compared using both the Richard-Campbell Sleep Questionnaire and the Restorative Sleep Questionnaire (Table 4), very good sleepers reported consistently better well-being compared to poor sleepers for daily, weekly, and monthly sleep (all *p* ˂ 0.001).

Somewhat stronger positive correlations with sleep quality were found using individual items taken from the VPS (Table 3). The item regarding time to fall asleep (long time – quickly) had a moderately strong relationship to daily, weekly, and monthly assessed with the Richards-Campbell Sleep Questionnaire, but only a weak relationship to daily, weekly, and monthly sleep assessed with the Restorative Sleep Questionnaire. The item regarding sleep quality (very poorly – well) also had a moderately strong relationship to daily, weekly, and monthly sleep assessed with the Richards-Campbell Sleep Questionnaire, but only a weak relationship to daily sleep assessed with the Restorative Sleep Questionnaire. In contrast, the item regarding participants’ feelings during the day (drowsy – rested) had a moderately strong relationship to daily sleep assessed with the Restorative Sleep Questionnaire, but only a weak relationship to weekly and monthly sleep. Finally, all correlations for this item were positive but weak for daily, weekly, and monthly sleep assessed with the Richards-Campbell Sleep Questionnaire.

### Stress

Correlations were stronger between the PSS-10 and sleep quality assessed with the Restorative Sleep Questionnaire than they were when sleep quality was assessed with the Richards-Campbell Sleep Questionnaire (Table 3). Specifically, there were moderate negative correlations between the PSS-10 and daily, weekly, and monthly sleep assessed with the Restorative Sleep Questionnaire, indicating that as stress increased, sleep quality decreased. However, there were only weak negative correlations between the PSS-10 and daily, weekly, and monthly sleep assessed with the Richards-Campbell Sleep Questionnaire. When sleep quality groups were compared using the Richards-Campbell Sleep Questionnaire (Table 4), very good sleepers reported significantly less stress compared to poor sleepers for daily (*p* = 0.019) and weekly (*p* = 0.002) sleep, but no differences were observed for monthly sleep. When sleep quality groups were compared using the Restorative Sleep Questionnaire, very good sleepers consistently reported significantly less stress compared to poor sleepers for daily, weekly, and monthly sleep (all *p* ˂ 0.001).

### Physical activity

There were no statistically significant correlations observed between sleep quality assessed with either the Richards-Campbell Sleep Questionnaire or the Restorative Sleep Questionnaire and physical activity assessed with the IPAQ-SF. All correlations were negligible (*r*_s_ ≤ 0.10) between daily, weekly, and monthly sleep quality and vigorous intensity, moderate intensity, and walking activities. When sleep quality groups were compared, there were no significant differences in vigorous intensity (*p* > 0.54), moderate intensity (*p* > 0.11), or walking (*p* > 0.36) activity between poor and very good sleepers for daily, weekly, or monthly sleep assessed using the Richards-Campbell Sleep Questionnaire. There were also no significant differences in vigorous intensity (*p* > 0.57), moderate intensity (*p* > 0.14), or walking (*p* ≥ 0.05) activity between poor and very good sleepers for any of the three time periods when sleep was assessed with the Restorative Sleep Questionnaire. Finally, when participants were grouped by total physical activity (high, moderate, low) for comparison of sleep quality assessed with the Richards-Campbell Sleep Questionnaire, there were no significant between-group differences in either daily (*p* = 0.631), weekly (*p* = 0.890), or monthly (*p* = 0.319) sleep quality. Non-significant differences were also observed for daily (*p* = 0.106), weekly (*p* = 0.425), and monthly (*p* = 0.445) sleep quality assessed with the Restorative Sleep Questionnaire.

### Summary

Table 5 provides a side-by-side comparison of the Richards-Campbell Sleep Questionnaire and the Restorative Sleep Questionnaire. Overall, both tools appear to perform similarly, with only minor differences, as evidenced by their similarity in total positive characteristics. Most importantly, both assess sleep similarly at each of the different time points. Furthermore, both discriminate between distinctly different groups of sleepers (very good vs. poor), although the Richards-Campbell Sleep Questionnaire may have some weaknesses in its ability to discriminate quality of life and stress for the 4-week (monthly) period, giving the Restorative Sleep Questionnaire a possible advantage for longer term assessment.

**Table 5.** Direct comparison of the Richards-Campbell Sleep Questionnaire and the Restorative Sleep Questionnaire.

	Richards–Campbell sleep questionnaire			Restorative sleep questionnaire		
Number of items	5			9		
Scale used	5-point			5-point		
Item scoring	5 items directly scored			6 items directly scored 3 items reverse scored		
	Day	Week	Month	Day	Week	Month
Assesses sleep quality similarly to comparison tool for same time period (day, week, month)	**+**	**+**	**+**	**+**	**+**	**+**
Discriminates differences in sleep quality between different time periods (day, week, month)	**+**	**+**	**+**	**+**	**+**	**+**
No floor effects observed	**+**	**+**	**+**	**+**	**+**	**+**
No ceiling effects observed	**-**	**-**	**+**	**+**	**+**	**+**
Discriminates differences in quality of life (RAND-36 vitality subscale) between poor and very good sleepers	**+**	**+**	**−**	**+**	**+**	**+**
Discriminates differences in well-being (VPS) between poor and very good sleepers	**+**	**+**	**+**	**+**	**+**	**+**
Discriminates differences in stress (PSS-10) between poor and very good sleepers	**+**	**+**	**−**	**+**	**+**	**+**
Discriminates differences in vigorous activity between poor and very good sleepers	**−**	**−**	**−**	**−**	**−**	**−**
Discriminates differences in moderate activity between poor and very good sleepers	**−**	**−**	**−**	**−**	**−**	**−**
Discriminates differences in walking between poor and very good sleepers	**−**	**−**	**−**	**−**	**−**	**−**
Has at least moderately strong correlation with time to fall asleep (single item)	**+**	**+**	**+**	**−**	**−**	**−**
Has at least moderately strong correlation with sleep quality (single item)	**+**	**+**	**+**	**−**	**−**	**−**
Has at least moderately strong correlation with feelings during the day (single item)	**−**	**−**	**−**	**+**	**−**	**−**
**Total positive characteristics**	**8**	**8**	**7**	**8**	**7**	**7**

The major limitation for the Richards-Campbell Sleep Questionnaire was the presence of ceiling effects for both the daily (sleep last night) and weekly (sleep during the past 7 days) versions. This limitation was not observed for any version of the Restorative Sleep Questionnaire. The major limitation for the Restorative Sleep Questionnaire appears to be the scoring algorithm that requires 3 out of 9 items (33%) to be reverse scored. This can be awkward and somewhat more time-consuming than directly scored items. Interestingly, the correlations with single-item questions regarding time to fall asleep and sleep quality favored the Richard-Campbell Sleep Questionnaire, while correlations with the single item regarding feelings during the day did not appear to favor either questionnaire.

## Discussion

The current study provides a comparison of two brief, self-reported tools that have previously been validated for assessment of sleep quality, the Richards-Campbell Sleep Questionnaire and the Restorative Sleep Questionnaire. Our goal was to identify an appropriate tool for use in busy clinical settings. To do so, we evaluated each tool’s assessment of sleep quality over three different time periods (last night, during the past 7 days, during the past 4 weeks) as well as each tool’s ability to discriminate between distinctly different types of sleepers (very good vs. poor) for a series of comparison measures (quality of life, well-being, stress, physical activity) that have previously been associated with one of the tools or another measure related to sleep quality.

Although we anticipated that the two tools might assess sleep quality very differently based on the populations originally used for validation (acute care vs. community) and the subtle conceptual differences between the two tools (perceptions of immediate sleep characteristics vs. feelings of being refreshed after waking), we were surprised to find no differences between sleep quality assessed with either the Richards-Campbell Sleep Questionnaire or the Restorative Sleep Questionnaire. Instead, our findings reflect equivalent assessment of sleep quality by both tools, with differences manifested only in relation to periods of time. For both tools, participants reported better sleep in the short-term (last night) and poorer sleep in the longer term (during the past 4 weeks). Furthermore, the number of women categorized as very poor, poor, good, and very good by each tool was remarkably similar.

One caveat relates to the Richards-Campbell Sleep Questionnaire and its inability to discriminate differences in quality of life and stress between good and poor sleepers solely over the longer term 4-week period. This discrepancy may be related to the conceptual nature of the tool and its focus on the immediate sleep experience. This could result in recall bias for the longer assessment period. However, the wording of the RAND-36 subscale used for quality of life measurement asks specifically about the “past 4 weeks,” while the PSS-10 used for measurement of stress asks specifically about “the last month,” so the lack of discrimination for the monthly version of the Richards-Campbell Sleep Questionnaire was unexpected. We are unable to provide a definitive explanation for this finding, which may be an anomaly and should not be considered an insurmountable barrier to use of the tool in clinical practice.

The ceiling effect observed for the Richards-Campbell Sleep Questionnaire daily and weekly versions is notable. None of the previous validation studies for either tool have evaluated floor and ceiling effects, which we believe is unfortunate, but consistent with the current state of the literature. Floor and ceiling effects are among the quality criteria recommended for evaluation of health status questionnaires.^79^ A recent systematic review of sleep tools identified 60 tools reported in 187 studies, yet only 9 studies adequately reported floor and ceiling effects.^47^ These varied widely between tools. Floor effects ranged from 0% to 28.3% and ceiling effects ranged from 0% to 76.4%.^47^ Given these wide ranges and sparsity of data, we are at a loss to interpret or explain our findings and would urge future studies to consistently report floor and ceiling effects.

In the current study, it is possible that the ceiling effects we observed for the Richards-Campbell Sleep Questionnaire were related to differences in study populations. The original validation study included hospitalized critical care patients who frequently experience impairments in overnight sleep quality due to pain, anxiety, noise, and medical devices.^80^^,^^81^ The questionnaire was subsequently validated in community-dwelling adults who reported significantly higher sleep quality for the previous night compared to intensive care patients.^52^ However, ceiling and floor effects were not reported. Given the higher sleep quality scores reported for the immediate overnight period by community-dwelling adults, there may be a greater likelihood of ceiling effects for short-term sleep assessment in that population using the Richards-Campbell Sleep Questionnaire.

The ability of both tools to discriminate between different categories of sleepers is an important finding, especially given the conceptual differences in how each tool measures sleep quality. The Richards-Campbell Sleep Questionnaire focuses on perceptions of the immediate sleep experience, while the Restorative Sleep Questionnaire focuses on feelings of being refreshed after wakening. In light of these subtle differences, perceptions of the immediate sleep experience could have resulted in different scores compared to feelings of being refreshed after sleep. This was not found to be the case and participants rated sleep quality similarly with both tools, and both tools discriminated equally between sleep categories.

Although it was not our primary study purpose, our findings provide some additional validation for both tools. Although known-groups validity is recognized as an important part of the validation process,^82^ we cannot find clear evidence that it was evaluated as part of the original validation of the Richards-Campbell Sleep Questionnaire.^49^ Known-groups validity has been reported for the Restorative Sleep Questionnaire^50^ but was limited to differences in mean scores for daily and weekly sleep quality between individuals with and without sleep disorders. In the current study, we extend that analysis to include comparison of participants based on the two extremes of sleep quality (poor vs. very good). As expected, very good sleepers reported better quality of life, greater well-being, and less stress compared to poor sleepers.

An unexpected finding was that our analyses were not influenced by the wording of the comparison measures regarding time periods. For example, the RAND-36 and PSS-10 ask specifically about feelings during the past month and so they might have been expected to have a stronger relationship with the questionnaire versions asking specifically about that time period. However, that was not found to be the case. The PSS-10 was found to be significantly related to sleep quality during all three time periods when assessed with either the Richards-Campbell Sleep Questionnaire or the Restorative Sleep Questionnaire, while the RAND-36 was related to the Restorative Sleep Questionnaire at all timepoints and only related to the Richards-Campbell Sleep Questionnaire for the 7-day time period. Although this may indicate a weakness for the Richards-Campbell Sleep Questionnaire, it was balanced by performance on the single items, and when quantified, the total number of positive features was similar for all three versions of both tools. Given the totality of our findings, we believe that both tools can be used for assessment of sleep quality based solely on clinical needs and preferences, although some caution may be needed for assessment over the shorter (daily, weekly) time period due to potential ceiling effects.

Another unexpected finding was the lack of association with physical activity. The bidirectional relationship between sleep quality and physical activity is well recognized, although the effect may be strongest for the next-day time period.^83^ However, we found no correlation with sleep quality at any of the three time periods evaluated. Moreover, moderate intensity activity has been found to have the greatest effect on sleep quality,^76^ yet we found no differences in sleep quality when vigorous intensity, moderate intensity, and walking activities were compared. We do not believe our findings were affected by wording of either the IPAQ-SF or the two sleep questionnaires. The IPAQ-SF asks specifically regarding activity during the past 7 days, which corresponds to the time period assessed by the weekly version of both sleep questionnaires. If the specific time period had exerted an influence on our findings, then at least a correlation with the weekly questionnaires would be expected. However, this was not the case. Instead, it is possible that our findings were influenced by the unusually high number of women reporting high levels of physical activity. Half of our sample reported greater than 3000 MET-min/week of activity. By comparison, population level data for women in the United States reflect a more balanced physical activity pattern with only 24% reporting high levels of activity, 29% reporting moderate levels, and 47% reporting low activity levels.^84^ Further research in this area is clearly needed.

A novel aspect of our study is our use of a 5-point scale for scoring the Richards-Campbell Sleep Questionnaire. Likert scales have previously been found to be comparable to VAS for subjective measurement.^62^ For example, when numeric rating scales and VAS are compared for subjective pain measurement, ratings are similar and use of a numeric rating scale is recommended primarily due to ease of use and greater compliance.^85^ Although use of a numeric scale instead of a VAS allowed a more direct comparison with the Restorative Sleep Questionnaire and provided what we believe to be a more efficient scoring process, it may have contributed to the questionnaire’s somewhat impaired ability to discriminate stress and quality of life between good and poor sleepers over the longer, 4-week period. As originally validated, the Richards-Campbell Sleep Questionnaire was intended only for overnight assessment, so this is the first study to evaluate its use for longer periods of time. The current findings provide support for its use beyond the immediate overnight period, as well as for use of the 5-point scale in lieu of a 100 mm VAS for scoring. Furthermore, we believe that the strong internal reliability statistics (Cronbach’s alpha), which ranged from .90 to .91 for both tools, support the validity of our findings and our use of the 5-point scale for the Richards-Campbell Sleep Questionnaire.

### Clinical utility

Overall, both tools have potential value for brief clinical assessment. Use of the 5-point scale for scoring provides precision of measurement as well as rapid scoring that minimizes burden for both patients and clinicians. The use of Likert scales for scoring is widespread and we believe the majority of adult patients are familiar with the 5-point scale. Also, our observation is that online assessments are being increasingly used to improve the efficiency of primary care visits and both tools lend themselves easily to an online format.

### Limitations

We recognize that there are limitations to our study. Although our sample reflected a broad age range, the average age was somewhat older (60 years) and it was limited to women only. Nevertheless, we believe our sample was representative of the primary care population in the United States. Older women represent a majority of adult primary care visits in the United States and primary care represents approximately 50% of all office visits in the United States.^86^ In addition, the prevalence of poor sleep quality reported in our sample was equivalent to the 13% population prevalence that has been calculated by meta-analysis for the United States, United Kingdom, and the Netherlands.^31^ Although our decision to include women only was based on recognized gender differences in sleep quality,^31^^,^^34^ and our decision to exclude current smokers was based on evidence regarding associations between smoking and sleep quality,^41^ we recognize that they limit the generalizability of our findings. Furthermore, we were unable to collect physiologic data to confirm hormonal status. This could have influenced our findings due to the association between hormonal status and sleep quality.^36^^,^^37^ Future studies are need with more diverse samples that address gender and racial differences, as well as hormonal differences between women of different ages.

We also did not evaluate sensitivity to change over time. Our study was cross-sectional and provided only a one-time measure for evaluation. The stability of sleep quality assessment between these questionnaires is an important clinical consideration and should be evaluated in future studies. Finally, due to the randomized nature of tool completion during data collection, we were unable to accurately measure the time needed for completion of the two questionnaires. Time is an important consideration that represents both patient and clinician burden. Although our primary interest in the current study was comparison of the two sleep quality assessments, we recommend that future researchers evaluate and report the time needed for questionnaire completion as well as scoring.

## Conclusion

Our findings do not strongly support the use of one sleep quality tool over the other.

For overall identification and assessment of good versus poor sleep quality, clinicians can choose either the Richards-Campbell Sleep Questionnaire or the Restorative Sleep Questionnaire. However, some caution may apply for short-term (daily, weekly) assessment for which the Restorative Sleep Questionnaire may be the more appropriate tool due to fewer ceiling effects.

## Data Availability

The data that support the findings of this study are available on request from the corresponding author. The data are not publicly available due to privacy or ethical restrictions.

## Transparency

### Declaration of financial/other relationships.

The authors have no relevant affiliations or financial involvement with any organization or entity with a financial interest in or financial conflict with the subject matter or materials discussed in the manuscript. This includes employment, consultancies, honoraria, stock ownership or options, expert testimony, grants or patents received or pending, or royalties. Peer reviewers on this manuscript have no relevant financial or other relationships to disclose.
